# Supplementary figures and images for: An optimized approach to germ-free rearing in the jewel wasp Nasonia
Source: PeerJ. 2016 Aug 9;4:e2316. doi: 10.7717/peerj.2316 (PMC4991892; doi:10.7717/peerj.2316)

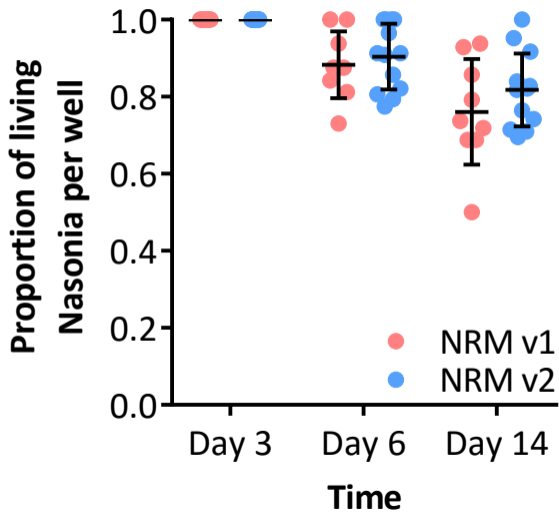

Supplement: Figure S1 — (A) The proportion of living Nasonia vitripennis in transwells on days 3, 6, and 14. There are no statistically significant differences in larval and pupal survival to day 14 on NRMv1 and NRMv2. R1, replicate one conducted by JDS; R2, replicate two conducted by EVO. Vertical bars with caps represent standard deviation from the mean. [file peerj-04-2316-s001.pdf]
